# Exploring access to care among older people in the last phase of life using the behavioural model of health services use: a qualitative study from the perspective of the next of kin of older persons who had died in a nursing home

**DOI:** 10.1186/s12877-015-0126-9

**Published:** 2015-10-26

**Authors:** Anna Condelius, Magdalena Andersson

**Affiliations:** Department of Health Sciences, Faculty of Medicine, Lund University, P.O. Box 157, SE-221 00 Lund, Sweden; City Office, Unit of Research and Development and Social Sustainability Development, Malmo, Sweden

**Keywords:** Health services accessibility, Aged/80 and over, Next of kin, Nursing homes, Qualitative Research

## Abstract

**Background:**

There is little investigation into what care older people access during the last phase of their life and what factors enable access to care in this group. Illuminating this from the perspective of the next of kin may provide valuable insights into how the health and social care system operates with reference to providing care for this vulnerable group. The behavioural model of health services use has a wide field of application but has not been tested conceptually regarding access to care from the perspective of the next of kin. The aim of this study was to explore the care accessed by older people during the last phase of their life from the perspective of the next of kin and to conceptually test the behavioural model of health services use.

**Methods:**

The data collection took place in 2011 by means of qualitative interviews with 14 next of kin of older people who had died in a nursing home. The interviews were analysed using directed content analysis. The behavioural model of health services use was used in deriving the initial coding scheme, including the categories: utilization of health services, consumer satisfaction and characteristics of the population at risk.

**Results:**

Utilization of health services in the last phase of life was described in five subcategories named after the type of care accessed i.e. admission to a nursing home, primary healthcare, hospital care, dental care and informal care. The needs were illuminated in the subcategories: general deterioration, medical conditions and acute illness and deterioration when death approaches. Factors that enabled access to care were described in three subcategories: the organisation of care, next of kin and the older person. These factors could also constitute barriers to accessing care. Next of kin’s satisfaction with care was illuminated in the subcategories: satisfaction, dissatisfaction and factors influencing satisfaction. One new category was constructed inductively: the situation of the next of kin.

**Conclusions:**

A bed in a nursing home was often accessed during what the next of kin regarded as the last phase of life. The needs among older people in the last phase of life can be regarded as complex and worsening over time. Most enabling factors lied within the organisation of care but the next of kin enabled access to care and contributed significantly to care quality. More research is needed regarding ageism and stigmatic attitudes among professionals and informal caregivers acting as a barrier to accessing care for older people in the last phase of their life. The behavioural model of health services use was extended with a new category showing that *the situation of the next of kin* must be taken into consideration when investigating access to care from their perspective. It may also be appropriate to include informal care as part of the concept of access when investigating access to care among older people in the last phase of their life. The results may not be transferable to older people who have not gained access to a bed in a nursing home or to countries where the healthcare system differs largely from the Swedish.

## Background

Ensuring that care is accessed on equal terms for the entire population constitutes a challenge to healthcare providers throughout the world. One group at risk of facing obstacles in accessing appropriate care when needed are those who live in nursing homes who are the oldest, most dependent and in the last phase of their lives. There is still little investigation into what care older people access during the last phase of their life and what factors that inhibit or enable access to various forms of care in this group. To examine this from the perspective of the next of kin may provide valuable insights into how the health and social care system operates regarding the provision of care for this vulnerable group.

A report from the European Commission [1] has revealed that older people do not access healthcare as frequently as they need and concludes that more research is needed to help tackle unequal access and the quality in care for older people. Particularly among those living in institutions. Previous research has shown a reduction in the utilization of hospital and ambulatory care among older people in the period following admission to a nursing home and that this reduction is on-going, despite their increased age and presumed deterioration in health over time [2–4]. Older people who live in nursing homes have been shown to utilize less hospital and outpatient care that those cared for at home, despite being older and having more health complaints [2]. This situation calls for more knowledge to be made available regarding access to care and factors that enable or hinders access in this group.

Several factors may enable or hinder access to care among older people. The majority of those admitted to a nursing home are physically and/or cognitively impaired making them dependent on others to manage everyday life [5]. The older persons’ own capacity to transform a need for care into a demand and to actually use healthcare services may constitute a hindrance to accessing care when it is needed. This hypothesis is supported by research showing that increased dependency in daily activities is negatively associated with utilization of healthcare services among older people [6]. Increased dependency on others puts the person in a vulnerable position where they have to rely on others to transform a need of care into actual use of care. This makes demands on a health and social care system where care needs among older and eventually dying people are detected and acted upon by others i.e. by professionals or by family members. Several factors within the provision and organisation of care may, however, contribute to hindering access to care by an older person. Poverty issues, organisational, geographical and transport problems, health literacy and health beliefs have been found to impact on the ability of dependent older people to access appropriate care [1]. A study by Muir-Cochrane et al. [7] reveals how the attitudes towards mental health problems among older persons and professionals and the availability of resources constitute barriers to accessing mental health services for older people.

Financial problems in combination with increased demand have given rise to a change in the provision of long-term care for older people in Europe and in Sweden, where more selective eligibility criteria for a bed in a nursing home have been applied [8, 9]. This has resulted in a higher level of dependency among those living in nursing homes, a heavier workload for staff and more end of life care is being provided in nursing homes [10, 11]. This requires staff in nursing homes to handle complex health and social care needs among residents and provide palliative care. Unfortunately poor communication, lack of care coordination, inadequate symptom control and the view among the staff that palliative care is not important have been found to constitute barriers to the implementation of palliative care in nursing homes [12].

A heavier work load for family members who provide care for their older relatives has also been seen during the recent decades. The significance of informal caregivers in the provision of long-term care for older people has received increase attention. Informal caregiving has been shown to be significantly related to the utilization of outpatient care [13], reduced Medicare expenditure for inpatient and long-term care [14] and reduced length of stay in hospital for older people [15]. Li [16] found that formal care does not replace informal care and that family members continue their care giving tasks even if formal care is available. Considering the ageing population and the increased reliance on informal caregivers it becomes important to investigate access and quality of care from the perspective of the next of kin.

### Theoretical model

Aday and Andersen [17] have developed a theoretical model for the investigation of access to healthcare services. The model was initially developed in the late 1960s in an attempt to add to the understanding of how and why families utilized health services [18] but has been modified several times after that. According to the model, use of services can be regarded as a human behaviour that is influenced by the social context in which it takes place. Consequently access to care is best evaluated by the actual use of services. “Utilization of healthcare services” constitutes the main component in the model alongside “Consumer satisfaction”. These two concepts are closely connected since satisfaction with the service utilized will probably impact on future use. “Characteristics of the population at risk” and “Characteristics of the healthcare delivery system” are other important components in explaining the use of health services [17]. According to the model a person’s utilization of health services is a function of his/her need for such services, a predisposition to use them and factors that enable them to be used. Enabling factors may be either characteristics of the person and include such aspects as insurance, regular source of care and income, or characteristic of the community where the person lives, including such as the availability of services. A person’s need refers to the severity of illness and is the factor most directly responsible for use. From the perspective of equality, access to care should be granted primarily according to need and not to other factors such as income or availability of resources [19].

The model has a wide field of application and has been used in studies focusing on the organisation and distribution of care and on the healthcare-seeking behaviour in various populations [20]. However, it has rarely been applied in qualitative research to illuminate access to care from the perspective of the next of kin and has not been conceptually tested with regard to their perspective. The aim of this study was to explore the care accessed by older people during the last phase of their life from the perspective of the next of kin and to conceptually test the behavioural model of health services use.

## Methods

### Sample

The sample consists of 14 next of kin of older persons who had died in a nursing home. This study is part of a larger longitudinal study described elsewhere [21]. The sample was identified retrospectively through a questionnaire sent to the next of kin to residents in nine nursing homes in Sweden in December 2009. The criteria’s for inclusion were that the next of kin had stated in the questionnaire that they were interested in taking part in an interview and that their older relative had died in 2010. 21 persons fulfilled these criteria’s and out of these seven dropped out since they lived in another part of the country (2), couldn’t be reached by telephone (2) or declined participation (3). Thus, 14 persons were eligible and included in the study.

Five were men and the relationships to the older person who had died were wife (1), brother (1), sister (1), daughter (6), son (3), daughter-in-law (1) and close friend (1). Four were in the age of 51–60, five were in the age of 61–65, four were in the age of 66–74 and one was in the age of 75–84. The older relatives had lived in the nursing home for approximately three and half years (approximated range 1–6 years).

### Data collection

Semi-structured interviews that were carried out in 2011 provided the data. The interviews were conducted 6–18 months after the older relative had died. Depending on their preference the interviews were carried out either in the participant’s home or at their working place. One interview was carried out with two next of kin simultaneously since this was what they preferred. Thus 13 interviews were conducted with 14 respondents. Six of the interviews were conducted by the first author (AC) and seven by the second author (MA) and all were audiotaped. Due to technical problems only half of one interview was recorded leaving approximately half of the interview unrecorded and thus unanalysed. The recorded interviews lasted for 21 (half interview) to108 min (mean 65 min, median 70 min).

An interview guide was used to ensure that all relevant aspects were covered. The interviews started with the open-ended question “Can you please tell me about the care received by your older relative during his/her last phase of life”? “The last phase of life” was defined by the respondents and no specific timeframe or definition was presented. This was done to capture the perspective of the next of kin and since there is no timeframe applicable to all older individuals. To illuminate the components “Characteristics of population at risk” and “Utilization of health services” the respondent were encouraged to talk about the care needs of their relative and how these needs were met and who initiated and who provided the care. To illuminate the component called “Consumer satisfaction” they were encouraged to talk about their satisfaction or dissatisfaction with the care received by their relative and the reasons for it. The guide was tested in two pilot interviews and minor revisions were made to clarify some of the questions.

### Qualitative analysis

All interviews were transcribed verbatim for analysis. The text from the interviews was analysed in several steps. The first step included reading the text as a whole to obtain an overall understanding of “what this is all about”. The authors did this independently and then came together to discuss their impression of the text. In the second step both authors read the text to identify meaning units related to the aim of the study and to sort out passages that were not relevant. Examples of passages in the text that was excluded were utterances regarding the time following the death of the older relative such as the arrangements for the funeral or the next of kin’s own situation and grief following the loss of a relative. In the next step the authors independently sorted the text inductively into rough categories related to the aim. The authors then met to compare and discuss their categorisations. These rough and somewhat unrefined categorisations corresponded well with the components in the theoretical model for the study on access [17], with some exceptions, and the work then moved on to the “main” analysis.

The interviews was now reanalysed using directed content analysis [22]. The conceptual model [17] was used in the derivation of the initial coding scheme and in the definition of the main categories. As can be seen in Table 1, the initial coding scheme consisted of three main categories derived directly from the model i.e., “Characteristics of the population at risk”. “Utilization of health services” and “Consumer satisfaction”. The category “Characteristics of the population at risk” contained the subcategories “Need” and “Enabling factors”, also derived from the model.

**Table 1:** Overview of the main concepts of the Behavioural model of health services use by Aday and Andersen [17] and the operational definitions of the concepts investigated in this study

Main concepts of the model		Operational definitions
Health policy *(not investigated)*	Characteristics of population at risk	Descriptions of the older person during the last phase of their life regarding:
	• Need	Health status and care needs
	• Enabling factors	Factors that enabled or hindered use of health services
	• Predisposing factors *(not investigated)*	
Characteristics of health delivery system *(not investigated)*	Utilization of health services	Descriptions of the care and services used by the older person during the last phase of their life
	Consumers satisfaction	Utterances regarding the next of kin’s satisfaction or dissatisfaction with the care accessed by the older person during the last phase of their life

In this step the two authors independently coded the meaning units according to the initial coding scheme. Any passage in the text that could not be coded according to the scheme was given a new code that covered its content. The authors then met to compare their categorisations and discuss the need for new categories and subcategories. Special attention was directed to the passages that had been given new codes and did not fit into the coding scheme. The next step included a constant movement between the whole and the parts, between the text and the codes, to identify patterns. The construction of subcategories and new categories was thus generated inductively, grounded in the text. The authors met to agree on new categories and subcategories. Both authors had a pre-understanding of caring for older persons as professionals (RN) and of research (PhD) but only one had any pre- understanding of the theoretical model.

### Ethical considerations

The Regional Ethical Review Board in Lund approved the study (Dnr 2009/527). All the respondents gave their written, informed consent to participation in the study.

## Results

### Characteristics of the older person

The main category C*haracteristics of the older person* comprises two subcategories named after the components in the model i.e. *Need* and *Enabling factors* (Table 2)*.* Both of these categories comprise subcategories that capture the internal variations in the needs or the enabling factors as they were told by the next of kin.

**Table 2:** Extended model with final categories and subcategories

			Added concept
Characteristics of the older person	Utilization of health services	Next of kin’s satisfaction	Situation of the next of kin
**Need** General deterioration Medical conditions and acute illness Deterioration when death approaches	**Admission to a nursing home** Access to basic care and social services Access to nursing care and medication	**Satisfaction**	**Feelings of remorse and insufficiency**
**Enabling factors** The organisation of care *The organisation of health and social care* *The organisation of care in the nursing home* *The organisation of care in ordinary housing* Next of kin The older person	**Primary healthcare**	**Dissatisfaction**	**Being dependent on the information given by the staff**
	**H** **ospital care**	**Factors influencing satisfaction** View of ageing and health Previous experiences	**Nursing home placement signifies security and less worry**
	**Dental care**		
	**Informal care** (added to the concept)		

Data in bold are subcategories that capture the main variation within each main category

Data in italic are subcategories that capture the internal variation in the subcategory “The organisation of care”

#### Need

When the next of kin were asked about the care received by their older relative during the last phase of their life, many started their story 1 or 2 years before their relative died. They had all observed deterioration in the relative’s health even before the move into the nursing home. The needs described by the next of kin are captured in three subcategories named *General deterioration, Medical conditions and acute illness* and *Deterioration when death approaches.*

##### General deterioration

The subcategory *General deterioration* describes a gradual deterioration and increase in the need for care and assistance in everyday life. Many suffered from cognitive impairment causing them to need supervision and guidance in daily activities. Physical impairments were also common such as pain and rigidity causing difficulties in walking and a need for aids and assistance. As many were very old they also suffered from the loss of relatives and friends causing a need for social stimulation. The needs described can be regarded as extensive and complex and becoming more serious with time. Below is a son’s description of his father while he was still living at home and how he became more confused and incapable of managing.

*It was when I noticed that he was losing his memory and forgetting words and other things too and physically there were no problems. He could walk and take a stroll. That was his, he’d always done that and he kept doing it. But he couldn’t find his way home and he couldn’t find, he couldn’t unlock the door with a key. That was how it started. And that was maybe a year before he went there (the nursing home). Then it got worse and worse and he was imagining a lot of things.* (Interview number 6)

##### Medical conditions and acute illness

This subcategory describes those conditions that the next of kin described as diagnoses or acute illnesses requiring more specific attention. Some of the older relatives experienced accidents or acute illnesses during the last phase of their life. These were described as various forms of fractures, infections, kidney stones, anemia and renal failure and pressure ulcers. These were also chronic conditions such as diabetes and Parkinson’s disease.

##### Deterioration when death approaches

The needs described in this subcategory were characterised by a considerable deterioration in health. The text mediated that the next of kin were often aware that their relative had now entered the final phase of their life and that death was near. Many observed a loss in their relative’s appetite and that they grew thinner and described them as just “skin and bones”. Some described how they became more reserved and uninterested in social contact and even “tired of life” or that the “spark of life was extinguished”. Those with cognitive impairment were seen as increasingly impaired, more confused regarding time and space and not recognizing their next of kin. In some cases the deterioration was triggered by an acute illness or trauma such as a fracture, an infection or loss of a close relative. Below a daughter narrates about her father and how he lost his appetite and body weight.

*No, he just kind of deteriorated. He was just skin and bones, skin and bones. And in the end he didn’t want to eat at all. Whiskey and peanuts were the last things that he wanted. I mean, that was what he ate.* (Interview number 10)

In the quotation below a daughter talks about her mother and how she became less interested in the world around her.

*Well, it was as if she had no joy left in her, kind of. That was how you could tell. She lost her spirit sort of and well, nothing was fun anymore and she got more and more, more and more scared of doing things. It was like she wanted to be in her own little closed of world.* (Interview number 11)

#### Enabling factors

Several factors were found to enable access to care in the last phase of life. These factors were sorted into three categories; *The organisation of care; Next of kin;* and *The older person*. These factors could also constitute barriers to accessing care when needed.

##### The organisation of care

The organisation of care appeared as central to the process of accessing it. When the organisation worked efficiently it meant that a need for care was detected and acted upon and care was accessed in relation to the needs. The organisation of care can be divided into an overarching organisation of health and social care and the organisation of the care in the nursing home or in the older person’s home. The subcategories are thus called: *The organisation of health and social care, The organisation of care in the nursing home* and *The organisation of care in ordinary housing.*

The narratives reflect well the organisation of the Swedish health and social care delivery system. A properly functioning organisation meant access to care when needed. The next of kin narrate how a hospital stay or a chronic condition meant follow-ups at the hospital or in primary healthcare. Many of the older persons gained access to a bed in a nursing home when they had been diagnosed with dementia. A contact with a family physician or a hospital stay lead to the detection of cognitive impairment, referral to a specialist and a diagnosis. This in turn lead to access to a bed in a nursing home. The stories also revealed how the organisation of health and social care could constitute a hindrance in accessing care when it was needed. Below a daughter in law tells how her mother in law could not get access to primary healthcare at home because obstacles within the organisation and to cooperation between various providers.

*And I asked the district nurse to go up there and run some tests on her. Because grandma couldn’t get down the stairs. The answer I got was that she’ll have to order a stretcher transport. I got so mad that I can’t describe it in words. I called the geriatric care people as she was their client and asked if they could do this for her, if they could get round this and use their influence over the health center, so they could work together. Not a chance, there was no cooperation between them whatsoever, was the answer I got.* (Interview number 12)

The organization of care at the nursing home was significant for the care accessed by the older residents. To move into a nursing home meant gaining access to staff around the clock and that registered nurses and primary physicians were within reach. It meant that meals were served three times a day and services, such as cleaning and laundry, were provided continuously. Various factors within the organization of care in the nursing home stood out as important for the care accessed. This is reflected in the five subcategories: *The organization of a contact person; The contact person as an individual; Staffing; Access to registered nurses and physician* and; *Routines at the facility.*

A contact person is an assistant nurse at the facility who was appointed as the person responsible for the provision of care and services to a specific resident. If this system functioned well it meant continuity of care. Thus laundry, purchases and daily care were carried out continuously and the next of kin were well informed. If the contact person knew the resident well this even meant they would notice changes in the resident’s health and notify a registered nurse or a physician and the next of kin. If the system did not function properly it meant the opposite i.e. that task were not carried out continuously, that the next of kin were not well informed and did not know who to turn to when they had questions. The narratives reveal that the residents and the next of kin had no influence over who was appointed as contact person or in the way in which this was organised at the facilities.

The text mediated that the contact person as an individual had a great impact on the care accessed by the residents, especially regarding care quality. The personal engagement of the contact person was important for the relationship with the resident, how tasks were performed and how contact with the next of kin functioned. Some contact people were described as “easy to talk to” and “trustworthy” while some were described as “tired” or “not genuine”. Those who were described in positive terms were seen as being more involved with the older person and their care, fulfilling their duties and keeping the next of kin informed. Those contact people who were described in negative terms did not fulfil their duties and were not regarded as trustworthy or genuine in their relation to the residents or to the next of kin.

Staffing at the facility was important for the care accessed by the residents. The narrations reveal that staffing around the clock signified that accidents or a change in health status could be detected within a reasonable space of time. It also meant that staff were available when needed and could provide care in accordance to the residents’ needs or wishes. If the staffing was insufficient it meant the opposite - that the needs of the residents were not met or the staff were not available when needed by the residents or by the next of kin.

Living in a nursing home meant having access to registered nurse around the clock. This in turn meant that the residents could be examined and treated for medical conditions at the facility or sent to the emergency room at the hospital when needed. The narratives revealed that physicians examined the residents when they were called for by the nurse.

The text revealed that there were certain routines at the facility that enabled access to care and palliative care when in the end of life. These routines included staff keeping vigil over the dying person and administration of morphine to reduce anxiety at the very end. Some participants said that they had had a conversation with the registered nurse at the facility when the relative moved in where the older person’s wishes about care were brought up. This was seen as important for the care, particularly when death was approaching. However, some relative also said that such a conversation never took place and that no one ever asked them or the older relative about their preferences or wishes concerning the care in the end of life.

*There’s this question, did you talk about the end at all, you and your sister, did you talk about that with your father? About the end, how he wanted it to be at the end?*

*We’d done that earlier, in connection with him moving to the nursing home and there the thing was that, the nurse there had talked about things during his registration interview. That nurse broached the subject of his future and when life ends and that…*

*You sat in a group together and talked about this?*

*Yes, yes.*

*Your father and you and her?*

*Yes, yes, how he wanted it when it happened and then we decided together that he should be in the nursing home for as long as it was possible. That was how we wished it would be. That’s not what happened, but it, that is what we wanted.* (Daughter talking about her father, Interview number 7)

All the older people had received some form of assistance before moving into the nursing home. This care was shown to be significant for accessing a bed in a nursing home. The narratives revealed how the staff in the home-help service were helpful in confirming that the situation was not functioning well and a placement in a nursing home was necessary. However, the next of kin also said that sometimes the organisation of home help did not work well causing problems, particularly in care quality.

##### The next of kin

The next of kin was significant for both when and the kind of care the older relative gained access to and also for the quality of care. The next of kin and other members of the family were very important for the social situation of their older relative. This was most evident in the very last days of life when they tended to be present more often at the nursing home. The next of kin also enabled the older person to remain at home despite extensive needs and contributed to their feeling of being secure at home. The next of kin often acted in their relative’s interests by contacting authorities or care providers in order to deal with problems or to obtain access to appropriate care, such as a bed in a nursing home or healthcare. The narratives reveal how the next of kin acted if there were mistakes or inaccuracies in the care of their relative and how they tried to correct them. Below a daughter explains how she reacted to the way her mother was treated at the facility.

*And then my father and I came, and they had just brought her in and put her facing a wall.*

*Left her there you mean?*

*Yes, and I reacted very strongly, and I said that even if she has very bad eyesight you don’t just push her against a wall, right. And then they shaped up afterwards but there were several times I kind of had to tell them to shape up…*

*Really?*

*…Oh yes, really, you don’t do that, right*. (Interview number 8)

It was also apparent that the next of kin were encouraged and invited to participate in decisions regarding medical examinations, treatments and even in life-sustaining care of their older relative. Below a woman tells how she, her daughter and the nurse at the facility had agreed that her demented husband should not receive any life-sustaining care.

*And then we had discussed it, both me and my daughter so, rather early on we told the nurse, we discussed with the nurse that, when there is a turn for the worse , care is to be provided but not life-support.*

*So you’d talked about that?*

*We had and that’s how it was.* (Interview number 3)

##### The older person

The will and attitude of the older person were significant for the care accessed, which is captured in the subcategory *The older person*. The attitude and will could either be positive in terms that he/she enabled access by accepting care or hindered it by refusing care. The next of kin told how their relative refused to accept more home help, refuses to visit their primary physician, the dentist, to move into a nursing home or even to participate in activities at the nursing home such as eating in the dining room or taking outdoor walks.

### Utilization of health services

The next of kin narrated about the care accessed by their older relative during the last phase of their life, often in relation to the need that caused the use of care. The main category *Utilization of health services* contains five subcategories named according to the type of care accessed: *Admittance to a nursing home, Primary healthcare, Hospital care, Dental care* and *Informal care* (Table 2).

#### Admittance to a nursing home

All of the older relatives had accessed a bed in a nursing home. For many next of kin the “last phase of life” comprises a time period of at least 2 years and they talked about the care accessed before and after their relative moved into a nursing home. Thus a bed in a nursing home was often accessed during what the next of kin regarded as the last phase of life. The care accessed in the nursing home was described in more detail that other care and thus constitutes the largest subcategory. Admission to a nursing home signified access to a variety of care and services which is included in the subcategories: *Basic care and services* and *Specific nursing care and medication.*

The nursing home placement implied that the older person had access to help and assistance around the clock and that they did not have to do their own cooking, cleaning, shopping or laundry. It also signified that they had access to care adapted to their needs and at the times they needed it. Many received help with daily activities such as toileting, getting dressed, showering and moving about. Even the food was adapted to the older person’s preferences, appetite and ability to chew. Assistance with feeding was given to those who could not manage by themselves. Walking aids or wheelchairs were used by those who had walking difficulties. The nursing home placement also meant gaining access to specific nursing care such as the bandaging of wounds and medication.

#### Primary healthcare

Next of kin also narrated that their relatives had contacts with a family physician at the nursing home and some had regular checkups at the primary healthcare center before moving into the nursing home. Below a son tells how a physician visited his mother at the nursing in order to treat a urinary infection.

*so, so it was like this, she came over there. One of them, a doctor who was in charge of the nursing home. Because she (the mother) had a lot of urinary infections and stuff for a while, so there was a lot with that, regarding that.* (Interview number 10)

#### Dental care

Some of the older persons accessed dental care on a regular basis. Here two brothers tell how their father got new denture.

*We forgot to tell you that they also took him to the dentist of course.*

*They did?*

*Yes, because he was so skinny, so his teeth became too large. He had dental plates since the age of thirty.* (Interview nr 4)

#### Hospital care

Those older relatives who had an accident or a serious illness during the last phase of their life were admitted to hospital and thus accessed specialized care. Below a daughter explains how her father accessed specialized care in hospital for urosepsis and renal failure about a year before he died.

*Then he got urosepsis and was admitted to hospital. He was in a bad way with his circulation. He basically had no blood pressure and he was, ehm, in very bad shape and we didn’t think he was going to make it. But they did all the lifesaving procedures on him and at this time he was 82 years old and, but they did all the procedures and he was put in ward x and was finally hooked up to a respirator and he was in ward x for seven weeks. For five of those weeks he was basically on a respirator and he was in a very bad way. His heart stopped a few times. However they resuscitated him there and ehm, he pulled through.* (Interview number 7)

#### Informal care

From the text it is apparent that the next of kin, other members of the family, friends and neighbours had provided care for the older person both when they lived at home and after their move to the nursing home. This referred in particularly to the social needs and stimulation of the older person but also to such services as looking after the older person’s finances, taking them for outdoor walks, driving them by car and making purchases.

*We were sometimes asked if they needed a different type of shoe. Of course, then we went shoe shopping for her at M (shopping centre) and drove the shoes out to her for her to try on.*

*You took quite a few shoes with you? Back to her home, yes?*

*Of course, because she couldn’t. Like, there was no point.*

*No, it’s difficult to try them on in that kind of surrounding.*

*The last thing we did for her was get her new glasses, in the beginning, when she moved in there (the nursing home). But that time we had to take her with us to M.*

(Interview number 11)

### Next of kin’s satisfaction

The next of kin expressed both satisfaction and dissatisfaction with the care accessed by their older relative, as is captured in the subcategories *Satisfaction*, *Dissatisfaction and Factors influencing satisfaction* (Table 2)*.*

#### Satisfaction

Satisfaction was strongly connected with their own feelings of safety, trust and confidence. If the next of kin felt confident that care was provided according to their older relatives needs and trusted the competence and kindness of the staff, it meant that they were satisfied. The relationship between the next of kin and the staff and in particular with the contact person was important for the feeling of trust. If the staff were perceived as friendly, trustworthy, genuine and caring towards the older person this contributed to the next of a-kin’s trust and satisfaction. When communication with the staff was seen as uncomplicated this also contributed to the feeling of trust and satisfaction with care.

*Was there any part of that care that you were particularly pleased with? How should I put this, how it was, what made it good there?*

*It was safe.*

*It was safe?*

*Mm*

*For you, for her?*

*For, well for her to, for the staff as well I think.* (Interview number 11)

#### Dissatisfaction

Just as the feeling of confidence and trust signified satisfaction with the care among the next of kin, lack of trust and confidence contributed to dissatisfaction. When the next of kin felt that the staff could not be trusted or when the staff failed to communicate with the next of kin this caused worry, mistrust and dissatisfaction with the care. The mistrust was often caused by negative incidences they had witnessed which put them on guard. If the care meant trouble and was a burden for the next of kin they expressed more dissatisfaction. Lack of out-door activities and social activities in the nursing homes were frequently mentioned as a causing disappointment and dissatisfaction. The transport service for disabled people was also a source of dissatisfaction.

#### Factors influencing satisfaction

The narratives reveal that the next of kin’s satisfaction with care was influenced by their own opinions, beliefs, expectations and previous experiences. This has been captured in the subcategories: *View of ageing and health* and *Previous experiences.* The next of kin tend to motivate their satisfaction or dissatisfaction with care with reference to the high age or the health status of their relative. Their narratives reveal beliefs that some symptoms, health complaints or disabilities can be regarded as a normal part of ageing and cannot or should not be treated. This means that they were satisfied with the care since they believed that no other care was beneficial or practicable because of the age or status of the older person. This point of view was expressed as the belief that more treatment or examinations “wouldn’t turn out well”, “would be wasted”, “pointless” or “cause more harm”.

*In retrospect, is there any kind of care that you wish he’d received, or was there anything missing?*

*No, not considering his condition.*

*No?*

*You have to think about it in regard to his status, so to speak (laughs). And I, I mean, he didn’t have, I cannot imagine that he would have got better if he had had all the stimulation in the world, or something. I don’t think so. Because it was an illness that progressed and, and there was only one possible outcome. So, no, I’m actually satisfied*. (Woman about her husband, Interview number 3)

Previous experiences and stories read in the newspapers or heard about from others gave rise to expectations regarding care and the next of kin compared the care accessed by their older relative to these experiences and expectations. The environment at the care facility, the staff, the treatment of the next of kin and the older person were all compared and judged this way. Satisfaction or dissatisfaction with the care was thus dependent on whether the previous experience was positive or not in comparison.

### The situation of the next of kin

This category was developed inductively and originated from the text, not from the theoretical model. When the next of kin narrated about the care accessed by their older relative during the last phase of their life they started from themselves and their own situation. The narratives reveal how closely related the next of kin are to their older relative and how these bonds impact their lives and their situation. This interrelationship is seen in the subcategories; *Feelings of remorse and insufficiency, Being dependent on the information given by the staff, Nursing home placement signifies security and less worry* (Table 2).

#### Feelings of remorse and insufficiency

Having an older relative in a nursing home led to feelings of remorse and insufficiency. Regrets about not having the time or the strength to take care of the person at home and “forcing” or “persuading” the person to move into a nursing home and not having the time to visit them more often were common.

#### Being dependent on the information given by staff

The next of kin lived with a more or less constant concern about the situation, the health and the wellbeing of their older relative. At the same time they could not be present all the time, which meant that they were dependent on the information given by their relative or the staff. If the relative suffered from dementia they were exclusively dependent on information given by the staff. The narratives reveal how the next of kin often were uninformed and left with their own ideas about the needs and the care of their relative, causing speculation and frustration. Most of the next of kin noticed that the health of their relative was getting worse and thought that death was approaching but received no confirmation of this from the staff at the facility until the very last weeks or days. When the next of kin was well informed about the care given and the status of their relative there were less frustration and worry. Below a woman tells how she had to rely on what the staff told her about the death of her husband.

*But I do understand. They can’t have staff sitting there hour after hour, can they. They say he sighed and I guess I’ll have to trust them, won’t I. He could have been dead for an hour, I don’t know, do I.* (Interview number 9)

#### Nursing home placement signifies security and less worry

Access to a bed in a nursing home for the older relative was a relief for most of the next of kin. The nursing home placement meant that they were no longer the main guardian and that the older person was looked after by others round the clock. Even though the next of kin experienced varying degrees of trust the nursing home placement generally signified a lessening of their burden. Here two brothers talks about the care of their father and how the situation with home care did not function well causing them worry about their father and how the care at the nursing home was perceived as safer and caused them less worry.

*Yes, there (at the nursing home) he had, I mean he was, he was fed four or five times a day. And there were people there all the time and he could push the button and then they would come right away. At home he could press the button but it could take a very, very long time before someone came. So that was, that feels a lot better for us.* (Interview number 4)

## Discussion

### Access to care during the last phase of life

The needs among older people in the last phase of life were shown to be complex and worsening over time with a more distinct deterioration closer to the end. The organisation of the health and social care system, the organisation of care at the nursing home and in ordinary housing, informal care and the older person were found to enable access to care when in the last phase of life. These factors could also constitute barriers to accessing care. The results reveal that older people access primary healthcare, hospital care, dental care and informal care during the last phase of their life and that a bed in a nursing home was accessed during what the next of kin regarded as the last phase of life. Satisfaction with care among the next of kin was strongly related to their feeling of safety and trust which in turn was connected to their relationship with the staff. The results showed how closely related the next of kin are to their older relative and how the situation and the care accessed by the older relative impact on their lives and situation.

All of the older relatives had died in a nursing home and thus had needed palliative care. This is supported by previous research showing that 27 % of those who access a bed in a nursing home die within the first year and 25 % in the second year [22]. The results reveal that some aspects of palliative care had been implemented in the nursing homes such as someone keeping vigil over the dying person and the provision of morphine to reduce anxiety at the very end. Some next of kin and their older relatives had even had a conversation with the nurse about the preferences of the older person regarding the care at the end of their life. This can be regarded as an acknowledgement that those who access a nursing home place are at the last phase of their life. However, the results also showed that the next of kin had noticed deterioration in the older person’s health even before the move into the nursing home and that they included this longer period of deterioration as part of the last phase of life. Many had also noticed when death was approaching but received no confirmation of this by professionals until the very last weeks or days. Thus, there seem to be a gap between what professionals and the next of kin regard as the last phase of life which may cause frustration and unmet needs among the next of kin and dissatisfaction with care. This shows the importance of a more holistic and family oriented approach in the care of older people where the experiences and the needs among the next of kin are taken into consideration.

The results reveal aspects of care in the nursing homes that need further attention. Many of the older persons were described as having lost their appetite and weight during their time in the nursing home and some were described as being only “skin and bones”. Some of the next of kin also said that their relative “was tired of life” and “wished to die”. According to the narratives, none of the older persons in this study had been examined regarding their nutritional state or depressed mood and none of them had accessed psychosocial support or counselling to help with possible grief or depression. Even if this result might be a reflection of the next of kin not having insight into all aspects of the care and thus cannot tell about the care accessed in relation to these needs this result is noteworthy. It may well reflect a view among staff where depressed mood, lost appetite and weight loss are seen as normal part of ageing and are not taken serious and are thus ignored. A study of the attitudes towards death and dying among staff in nursing homes reveals that 63 % regard depression as a normal part of older people dying and 26 % regard depression as untreatable at the end of life [23]. However, to distinguish between grief and depression in people who are dying may be difficult since the diagnostic symptoms for depression are often present in people feeling grief because they are preparing for death [24]. Voluntarily stopping of eating and drinking (VSED) is a process in which a cognitively intact and competent person voluntarily and deliberately chooses to shorten their life by stopping eating and drinking and can be regarded as an expression of autonomy and control [25]. It is doubtful that the occurrence of the nutritional problems described among the older relatives in this study can be explained as their making a deliberate choice to shorten their life. Particularly since many suffered from dementia. Weight loss among older people in nursing homes has been shown previously to be significantly associated with depression, poor oral intake, swallowing issues and eating/chewing dependency [26]. All these factors, but depression in particular, are serious and should be treated as a part of professional care. Access to care among older people in nursing homes in relation to nutritional problems, depressed mood and grief needs further investigation. In particular regarding factors acting as barriers to accessing care in relation to these needs.

Yet another striking result was that the next of kin were invited by healthcare staff to participate in important decisions regarding medical examinations, medical treatments and even life-saving interventions, sometimes without the involvement of the older person. Even if the next of kin act with the best intentions and in, what they believe, is the best interest of their relative, this conduct is questionable. This is particular so since the results also reveal ageist views among the next of kin, in that they perceive some conditions and complaints as natural part of ageing with further examinations or treatments being regarded as “pointless” or even “wasted”. These results are in line with those from an ethnographic study of ageism and stigma in residential care for older people conducted by Dobbs et al. [27]. Their study revealed an ageist worldview among family members, staff members and among older residents and that decisions regarding care and treatment were made with family members with no input from the older person him/herself. Decisions regarding medical and life-saving interventions among older people are problematic since there is limited evidence that medical interventions do not benefit older people, due largely to that they are excluded from clinical trials [1]. There is a thin line between acting in a person’s best interest and violation their autonomy. This is particularly true when there are ageist views present among those who decide what care the older person should have access to i.e. among family members and those who provide care, without input from the older person him/herself. More research is needed regarding stigmatic and ageist attitudes as a barrier to accessing care for older persons in the last phase of their life since no firm conclusions can be made from this study.

The next of kin’s satisfaction or dissatisfaction with the care was strongly linked to their feelings of trust or mistrust of the staff. This is in line with a study by Hasson and Arnetz [28] about care recipients’ and family members’ perception of the quality of care for the elderly showing that professional skills of the staff, contact and care were significant predictors of relatives’ overall quality ratings. The next of kin were shown to be left with their own thought and speculations about the situation, the health status and needs of their older relative which caused frustration and even dissatisfaction with the care. A review by Andershed [29] focusing on the situation and needs of relatives in end of life care found a similar result. The review demonstrated a need for information and communication among relatives about the patient’s condition, symptoms and treatments and available resources and showed that the more relatives understood about care the more satisfied they were. Thus, information, communication and the attitudes of the staff are decisive in relation to satisfaction with care among the next of kin.

### Modification of the behavioural model

One aim of this study was to conceptually test the behavioural model of health services use with regard to the perspective of the next of kin. According to the model access is defined as entry to the healthcare delivery system [17] and informal care is thus not included. When it comes to access to care among older people in the last phase of their life it may, however, be appropriate to include both formal and informal care in the concept. The results showed that the next of kin and other informal caregivers provided care and services for their older relative and contributed significantly to quality in care and enabled access to various forms of care. Sometimes they were even involved in and made important decisions regarding medical examinations, treatments and lifesaving interventions for their older relative. This result is supported by previous research showing that informal caregivers provide significant amount of care for older relatives [16] and enables access to the healthcare system [30]. Even though it has not been definitely established whether informal care constitutes a substitute or a complement to formal care the increased significance of informal caregiving in the long-term care for older people cannot be overseen. Johansson, Long and Parker [31] view the increased interest and focus on informal caregiving for older people in Sweden as a plausible paradigm shift to a more family oriented welfare system. This shift may however be problematic in terms of equality since many haven’t got access to informal caregivers. Further research is needed to evaluate possible inequalities in access and quality in care among older people with or without access to informal caregivers.

The behavioural model was extended with the new category named “*The situation of the next of kin”.* This category makes it clear that if access to care is to be investigated and understood from the perspective of the next of kin their situation must be taken into consideration. The next of kin were closely involved in the care of their older relative, by providing care, enabling access to various forms of care and by being on guard against any mistreatment. Andersson et al. [32] found a similar result in their study about the experience of being the next of kin to an older person in the last phase of their life. Their study showed that being a next of kin meant being responsible for the relative and needing to be acknowledged by the professionals. What this study adds is the knowledge that the care accessed by the older relative impacts on the situation and the satisfaction with the care among the next of kin. It is obvious that if the care accessed by the older relative put the next of kin in a stressful situation and caused them worry and discomfort, this signified dissatisfaction with care. This does not, however, have to mean that the care accessed by the older person is inappropriate in relation to their needs but, is rather, inappropriate in relation to the needs of the next of kin. This is an important distinction that must be taken into consideration when investigating access and quality in care from the perspective of the next of kin or other proxies.

### Methodological considerations

There are limits to this study that need to be addressed. The data collection took place by means of qualitative interviews with 14 next of kin of older people who had died in a nursing home in Sweden. Thus, the subcategory named “*Admittance to a nursing home*” is primary a result of the sampling procedure and the results may not be transferrable to older people who have not gained access to a bed in a nursing home or to countries where the healthcare system differs largely from the Swedish.

Whether the next of kin can be regarded as a reliable source of information when investigating access to care is open for discussion. According to the results in this study the next of kin are closely involved in the care of their older relatives and can be regarded as a vital source of information after the older person has died. A review by McPherson and Addington-Hall [33] suggest that proxies can be reliable in reporting the quality of services and on observing symptoms in the end of life care but their reports on aspects of the patient’s subjective experiences should be interpreted more carefully. In this study it is evident that the next of kin did not and could not have insight into everything that happened to their older relatives regarding their health, wellbeing, care or treatments. Since the interviews were conducted 6–18 month after the older person had died there is also a risk of recall bias. This puts limits the conclusions that can be made from this study regarding actual access in relation to needs among older people. The results should not be regarded as a comprehensive coverage of the older person’s needs or the care accessed in relation to those needs. However, considering the greater reliance on informal caregivers in the provision of care for older people their involvement, perception and understanding of care become increasingly important.

The analysis was performed using directed content analysis [22]. The model for investigation of access to healthcare services developed by Aday and Andersen [17] was used in the derivation of the initial coding scheme. The use of the initial coding scheme in the sorting and categorization of data enhances reliability and maximize agreement between coders [34]. The risk in this approach is that the analysis becomes biased by the theory and that results that do not support the theory are not regarded as relevant and are excluded. For this reason an initial, naïve categorisation of data was performed without applying the coding scheme and special attention was paid to the passages in the text that could not be categorised according to the theory. The derivation of a new category demonstrates the main advantage of using directed content analysis is that an existing theory can be supported and extended [22].

## Conclusions

A bed in a nursing home was accessed during what the next of kin regarded as the last phase of life and the needs among the older relatives were varied, complex and sometimes extensive. Specialised hospital care was accessed for acute illnesses or traumas. Physicians and registered nurses enabled medical treatments and examinations to be performed at the nursing home. Some aspects of palliative care were implemented in the nursing homes. More attention is, however, needed to be given to nutritional state and depressed mood among residents in nursing homes and the care accessed in relation to these particular needs. More research needs to be directed to ageism attitudes among health professionals and informal caregivers acting as a barrier to accessing care for older people in the last phase of life. The next of kin enable access to care and contributed significantly to care quality for their older relatives. The theoretical model for access to health services was extended with the addition of a new category *The situation of the next of kin* - making it clear that their situation must be taken into consideration when investigating access to care from their perspective. The care accessed by the older relative impacts on the situation and the satisfaction with the care among the next of kin. If informal caregivers are to continue to provide care to their older relatives and enable access to care it is essential that they receive the support and relief they require. The results from this study may not be transferable to older people who have not gained access to a bed in a nursing home or to countries where the healthcare system differs largely from the Swedish.
